# Arc-Induced Long-Period Fiber Gratings at INESC TEC. Part II: Properties and Applications in Optical Communications and Sensing

**DOI:** 10.3390/s21175914

**Published:** 2021-09-02

**Authors:** Gaspar Rego, Paulo Caldas, Oleg V. Ivanov

**Affiliations:** 1 ProMetheus, Instituto Politécnico de Viana do Castelo, Rua Escola Industrial e Comercial Nun’Álvares, 4900-347 Viana do Castelo, Portugal; pcaldas@estg.ipvc.pt; 2 Center for Applied Photonics, INESC TEC, Rua Dr. Roberto Frias, 4200-465 Porto, Portugal; 3 Ulyanovsk Branch of Kotel’nikov Institute of Radio Engineering and Electronics of Russian Academy of Sciences, Ulitsa Goncharova 48, 432071 Ulyanovsk, Russia; olegivvit@yandex.ru; 4 Ulyanovsk State University, Ulitsa L. Tolstogo 42, 432017 Ulyanovsk, Russia

**Keywords:** long-period fiber grating, arc-induced fiber grating, optical communications, optical fiber sensor

## Abstract

In this work, we review the most important achievements of INESC TEC related to the properties and applications of arc-induced long-period fiber gratings. The polarization dependence loss, the spectral behavior at temperatures ranging from cryogenic up to 1200 °C and under exposure to ultraviolet and gamma radiation is described. The dependence of gratings sensitivity on the fabrication parameters is discussed. Several applications in optical communications and sensing domains are referred.

## 1. Introduction

Long-period fiber gratings (LPFGs) exhibit resonances at specific wavelengths as a result of coupling of the core mode to discrete cladding modes and can work as wavelength selective optical filters. Therefore, LPFGs find application in the optical communications domain as band rejection filters. They have been used or proposed for a number of applications such as gain equalization in erbium doped fiber amplifiers, elimination of Stokes orders in cascaded Raman amplifiers/lasers, suppression of the stimulated Raman scattering in MOPA fiber laser and the amplified spontaneous emission [1,2,3,4,5,6,7,8]. They have also been used for temperature stabilization of Er-doped superfluorescent fiber sources [9]. The gratings’ rejection bands can be made very broad [10] being useful for polarization dependent loss compensation. Ultimately, they can be made wavelength independent and, consequently, can act as variable optical attenuators [11]. On the other hand, by cascading two identical gratings it is possible to obtain very narrow optical filters [12,13,14] being, therefore, useful devices in wavelength division multiplexing and optical code division multiple access systems [15,16]. The use of LPFGs for dark hollow beam generator [17], or as mode converters in standard fibers [18], two-mode fibers [19,20] and as wavelength selective polarizers [21,22,23,24,25,26] has also been demonstrated. LPFGs can be used to enhance coupling between fiber and a waveguide [27], a semiconductor laser or a bulk optical element [28]. Coupling between a fiber and the free space by producing a planar wave was also demonstrated [29]. Two important applications of LPFGs in optical communications such as optical switches [30,31,32] and add-drop multiplexers can be realized by using two gratings in close proximity [33], a grating assisted fiber coupler [34] or coupling between mismatched twin-core fibers [35]. LPFGs can act as ultrafast optical differentiators [36]. The use of LPFGs as dispersion compensators has also been investigated theoretically [37,38,39]. LPFGs alone or with other gratings have been used for producing multiwavelength fiber lasers [39,40,41,42,43] or simply a more stable fiber laser output [44]. LPFGs can also act as bandpass filters by using phase-shifted gratings [45,46] or two concatenated gratings with a core-mode blocker in between [47,48,49,50].

Long period fiber gratings are very sensitive to changes in physical parameters, such as, temperature [51], strain [52,53], transverse load [54], bending [55], torsion [56] and refractive index of the surrounding medium [57,58,59] and, therefore, several sensor heads have been proposed to monitor temperature in extreme environments [60,61], structural integrity [62], multi-directional bending [63] and torsion [64]. On the other hand, coated LPFGs [65,66,67] can act as refractometric sensors [68,69] able to monitor an enormous quantity of physical [70,71,72], chemical [73,74,75] and biological parameters [76,77,78,79,80].

In the following sections we will present the properties of arc-induced gratings and discuss several applications in the sensing field. Since one of the first identified advantages of LPFGs produced by using the electric arc technique is their stability at high temperatures, we begin by presenting a detailed study on the thermal behavior of these gratings. Thus, we introduce the equations related to the determination of gratings sensitivity, followed by the thermal behavior up to 1200 °C, the effect of the heating cycles namely on the strain point, the study of the stability at high temperatures over time and finally their behavior at cryogenic temperatures. In the third section we discuss the influence of the fabrication parameters on the temperature and strain sensitivity, followed by their sensitivity to other physical parameters such as, bending and surrounding refractive index. Afterwards, we present an important characteristic of arc-induced gratings: they intrinsically exhibit high polarization dependence loss (PDL) [81], which in fact imposes limitations on their use in the optical communications domain. On the other hand, this property also enables their use as a sensor for the simultaneous measurement of temperature and strain [82]. The section ends by showing the effect of LPFGs exposure to uniform uv-radiation and gamma radiation. The first gives information related to the mechanisms of gratings formation and the second related to the possibility of using sensors, based on arc-induced gratings, in extreme environments. The fourth section is concerned to the study of three sensor heads that allow for the simultaneous measurement of temperature and strain, that overcome cross-sensitivity and finally an original flow sensor is also presented.

## 2. Thermal Behavior of Arc-Induced Gratings

Among the different physical parameters that gratings can be used to monitor, temperature is one of the most important. Therefore, a detailed analysis on their thermal behavior will be presented ranging from cryogenic up to very high temperatures. Naturally, the behavior depends on the fiber itself.

### 2.1. Theoretical Equations Concerning the Gratings Sensitivity

For an LPFG, the wavelength at which the mode coupling occurs is given by [83]:

(1) $$\lambda_{res}=\left(n_{co}^{eff}-n_{cl,m}^{eff}\right)\Lambda$$

where *λ_res_* represents the resonance wavelengths, *Λ* is the grating period and $n_{co}^{eff}$ and $n_{cl,m}^{eff}$ are the effective refractive indices of the core mode and the *m*-th cladding mode, respectively.

The temperature sensitivity of an LPFG is expressed by the following equations [84]:

(2) $$\frac{d\lambda_{res}}{dT}=\lambda_{res}\gamma \left(\alpha +\Gamma_{Temp}\right)$$

where *α* is the thermal expansion coefficient of the fiber. *γ*, the general sensitivity factor, describes the waveguide dispersion and is expressed by:

(3) $$\gamma =\frac{\frac{d\lambda_{res}}{d\Lambda }}{n_{co}^{eff}-n_{cl,m}^{eff}}$$

where *dλ_res_*/*dΛ* represents the slope of the dispersion curves. Γ*_Temp_* describes the temperature dependence of the waveguide dispersion and is expressed by:

(4) $$\Gamma_{Temp}=\frac{\xi_{co}n_{co}^{eff}-\xi_{cl}n_{cl,m}^{eff}}{n_{co}^{eff}-n_{cl,m}^{eff}}$$

where *ξ_co_* and *ξ_cl_* are the thermo-optic coefficients of the core and cladding materials.

Analogously, the strain sensitivity can be expressed by:

(5) $$\frac{d\lambda_{res}}{d\varepsilon }=\lambda_{res}\gamma \left(1+\Gamma_{strain}\right)$$

Γ*_strain_* describes the strain dependence of the waveguide dispersion and is expressed by:

(6) $$\Gamma_{strain}=\frac{\eta_{co}n_{co}^{eff}-\eta_{cl}n_{cl,m}^{eff}}{n_{co}^{eff}-n_{cl,m}^{eff}}$$

where $\eta_{co}$ and $\eta_{cl}$ are the elasto-optic coefficients of the core and cladding materials.

In which concerns the surrounding refractive index sensitivity, the following equations apply:

(7) $$\frac{d\lambda_{\mathrm{res}}}{dn_{\mathrm{surr}}}=\lambda_{\mathrm{res}}\gamma \;\Gamma_{\mathrm{surr}}$$

where Γ*_surr_* is expressed by:

(8) $$\Gamma_{surr}=-\;\frac{u_{m}^{2}\lambda_{res}^{3}n_{surr}}{8\pi r_{cl}^{3}n_{cl}\left(n_{co}^{eff}-n_{cl,m}^{eff}\right){\left(n_{cl}^{2}-n_{surr}^{2}\right)}^{3/2}}\;\left(\mathrm{valid}\;\mathrm{for}\;n_{surr}<n_{cl}\right)$$

and describes the dependence of the waveguide dispersion on the surrounding refractive index (*n_surr_*). The term *u_m_* is the *m*-th root of the zero-order Bessel function and the other symbols have their common meaning. A discussion on the ways to increase the LPFGs sensitivity to changes on the surrounding refractive index was presented in [68]. The analysis of the above equations enables one to conclude that changes to the effective refractive indices of core and cladding will impact the gratings sensitivities. In particular, as will be shown below, since the arc discharge affects not only the fiber cross section but also changes the strains in the fiber core and cladding it is expected that the temperature and strain sensitivity may be tuned by adjusting the fabrication parameters.

### 2.2. LPFGs’ Temperature Sensitivity from Room Temperature up to 1200 °C

First, we present the behavior from room temperature up to 1200 °C for gratings arc-induced in different type of fibers and afterwards we will present the behavior at cryogenic temperatures.

**Figure 1:**
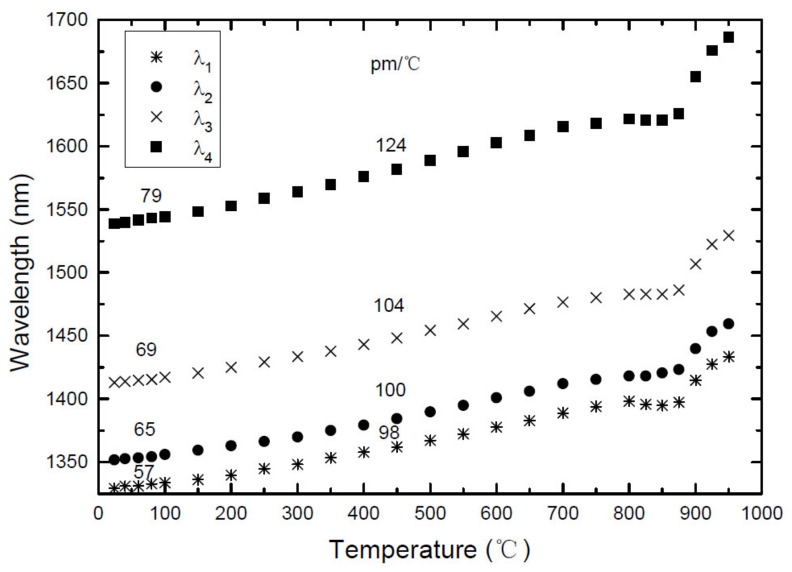
Thermal behavior of a 540 μm LPFG arc-induced in the Siecor fiber. Reprinted with permission from ref. [85]. © 2021 Taylor and Francis.

In order to investigate the gratings thermal behavior, they were placed inside a tubular oven and a weight was applied to keep the fiber straight. The temperature was raised from room temperature up to about 1000 °C in steps of typically 50 °C. At each step the temperature was kept constant for about 10 min in order to the fiber reach thermal equilibrium. Figure 1 shows the general behavior for a 540 μm LPFG written in the Siecor fiber. As it can be seen the resonance wavelengths shift non-linearly towards longer wavelengths as the temperature increases. The temperature sensitivity increases with the order of the cladding mode resonances and also with the temperature increase except around the strain point.

**Figure 2:**
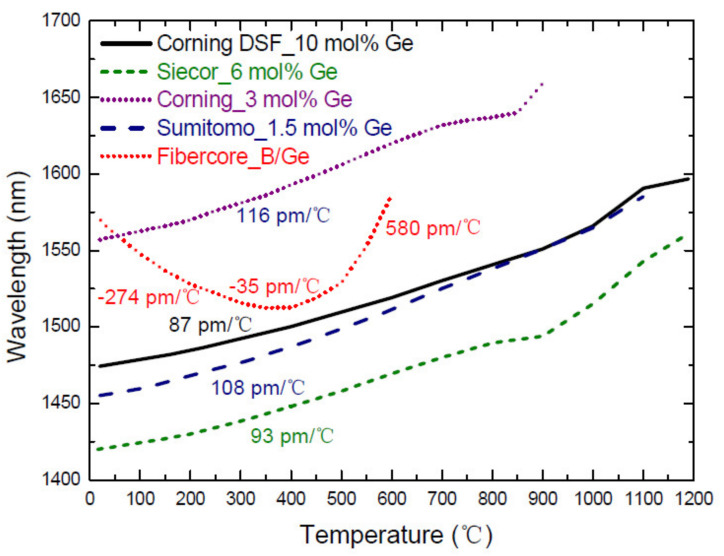
Thermal behavior of LPFGs arc-induced in Ge-doped fibers. Reprinted from [86].

**Figure 3:**
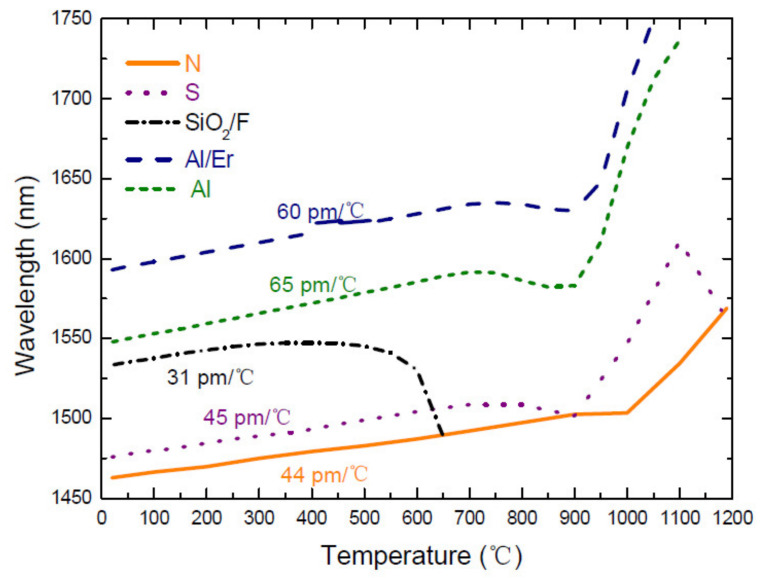
Thermal behavior of LPFGs arc-induced in Ge-free fibers. Reprinted from [86].

The thermal behavior of LPFGs arc-induced in Ge-doped fibers and in Ge-free fibers is presented in Figure 2 and Figure 3, respectively. In general, for Ge-doped fibers the resonance wavelengths shift quadratically with the temperature increase from room temperature up to the strain point. At this point, typically around 700 °C, the glass structure starts to relax and depending on the fiber composition and fabrication history (drawing temperature and tension) the resonance wavelengths show different behaviors, that is, the slope decreases or even become negative as will be discussed below. For the B/Ge co-doped fiber the resonance wavelengths shift towards shorter wavelengths up to 300–400 °C and afterwards to longer wavelengths. This is a consequence of the presence of B_2_O_3_ that decreases the thermo-optic coefficient in comparison to the one of SiO_2_ and GeO_2_. Afterwards, the slope becomes more pronounced due to the thermal expansion of glass, since its contribution becomes comparable to the dependence of the refractive index on temperature.

**Figure 4:**
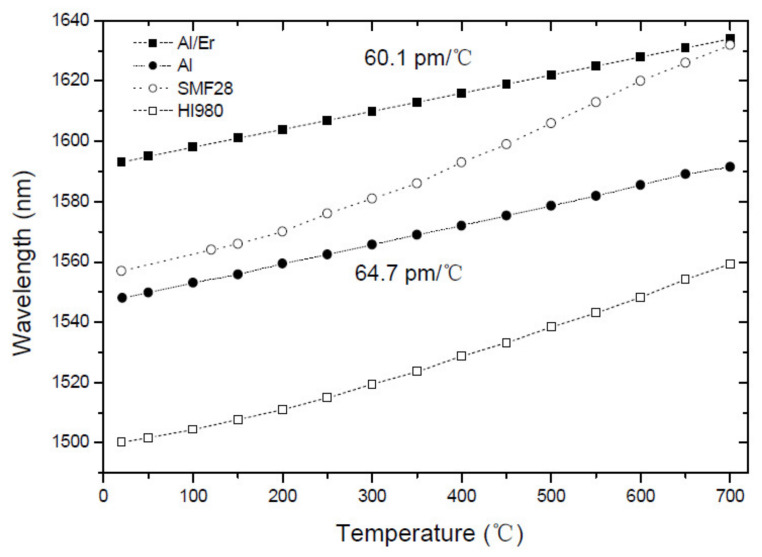
Comparison of the thermal behavior of LPFGs arc-induced in Ge-doped and in aluminosilicate fibers. Reprinted from [86].

The major difference between these two figures is that the temperature sensitivity is constant for Ge-free fibers while it shows a linear dependence for Ge-doped fibers. This behavior becomes more evident in Figure 4. Table 1 summarizes the temperature sensitivities obtained for gratings inscribed in different fibers. Thermal studies below 200 °C were also conducted in Ge-free fibers by S. Campopiano et al. [87,88,89]. In the case of pure-silica core fibers and Er doped fibers similar results were obtained. For gratings inscribed in a P-doped fiber, values for the temperature sensitivity ranging from −58 to −116 pm/°C were obtained (also temperature dependent) for two distinct grating periods and cladding mode orders.

### 2.3. Heating Cycles and Strain Point

**Figure 5:**
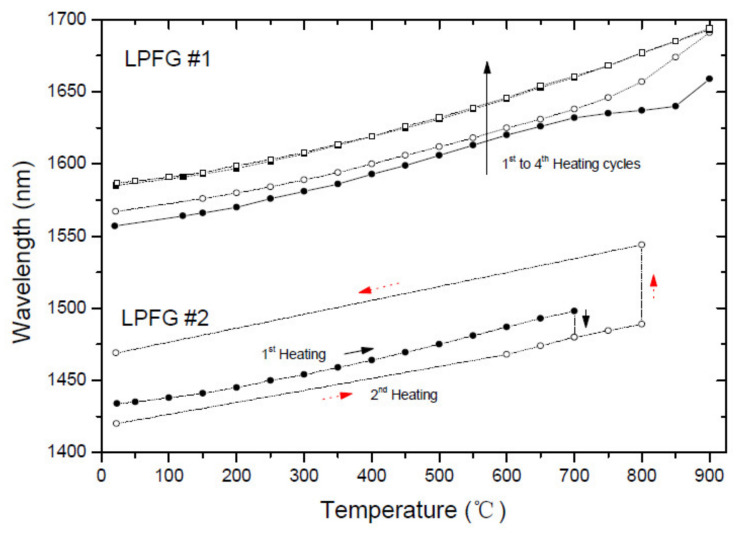
Thermal behavior of LPFGs submitted to several heating cycles. Adapted with permission from ref. [85]. © 2021 Taylor and Francis.

The thermal behavior can be modified by submitting the LPFGs to heating cycles. One grating (fabrication parameters: 5.1 g, 9 mA, 1 s, 40) was heated from room temperature up to 900 °C four times and as Figure 5 shows after the third annealing the fiber was in thermal equilibrium (the fourth cycle retraces the third one). As it can be seen, comparatively to the first heating cycle the whole spectrum shifted towards longer wavelengths and the stress relaxation region vanished. The figure also shows the annealing of a second similar grating, but this time the grating was heated up to 700 °C and was kept at that temperature for 15 h. It was observed that the LP_13_ cladding mode shifted 18 nm towards shorter wavelengths (LP_14_ shifted 25 nm). It should be stressed that the LPFGs fabrication parameters may have some influence on this behavior since a third grating (1.1 g, 12 mA, 0.5 s, 40) shown a down shift of 55 nm. Moreover, detailed studies were presented by Humbert et al. [90,91] in the 700–800 °C temperature range concerning gratings fabricated with no use of external tension and exhibited lower wavelength shifts. After cooling down the second grating to room temperature, it was submitted to an annealing temperature of 800 °C for 6 h. This time the spectrum moved 55 nm towards longer wavelengths. This may reflect a rearrangement of the glass structure towards a new thermodynamic equilibrium as a consequence of the interplay between stress relaxation and the fiber thermal expansion coefficients and viscosities, which are also time and temperature dependent.

**Figure 6:**
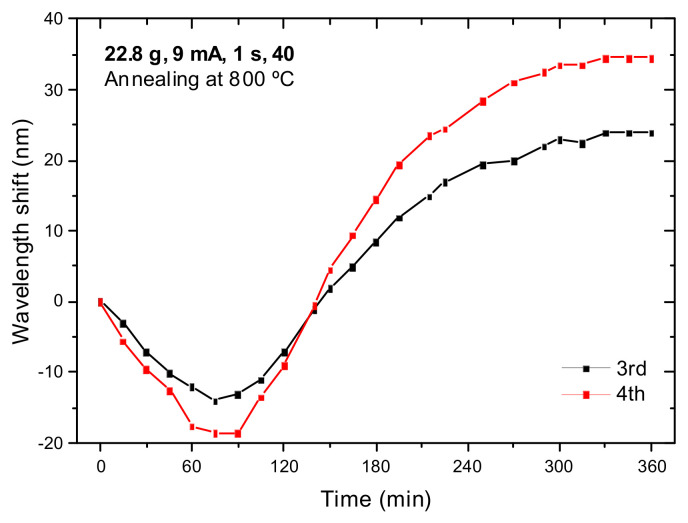
Time dependence of two resonances during annealing at 800 °C for 6 h. Reprinted from [86].

Stress relaxations in the 700–800 °C temperature range was first observed in [92]. Figure 6 shows the movement of two resonances belonging to a LPFG (22.8 g, 9 mA, 1 s, 40) during the annealing at 800 °C for 6 h. As it can be observed, the resonances move first towards shorter wavelengths and afterwards towards longer wavelengths where they stabilize, being the shift larger for the highest order cladding mode.

**Figure 7:**
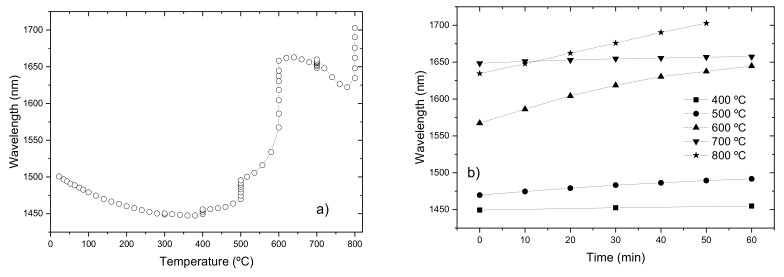
(**a**,**b**) Heating cycle up to 800 °C with dwell time of 1 h at each step. Reprinted with permission from ref. [96]. © 2021 Wiley Periodicals, Inc.

However, stress relaxations can occur at much lower temperatures, around 300 °C as was found for pure-silica-core fibers and for the FiberCore B/Ge co-doped fiber [93,94]. Relaxations at about 400 °C were also found in elongation experiments with several types of fiber drawn with different drawing tensions [95]. Figure 7 shows the thermal behavior of LP_05_ cladding mode belonging to a LPFG (5.1 g, 9 mA, 0.5 s, 400 μm, 60) arc-induced in the B/Ge co-doped fiber. A minute change can be detected at 300 °C but it increases at higher temperatures. After cooling down the LPFG from temperatures in the range of 300–700 °C, the LP_05_ cladding mode shifted to longer wavelengths by 2, 12, 50, 203 and 249 nm, respectively. It should be noted that the erasure of the grating started for temperatures above 700 °C, being completely eliminated after 25 min at 800 °C.

### 2.4. LPFGs’ Stability at High Temperatures

**Figure 8:**
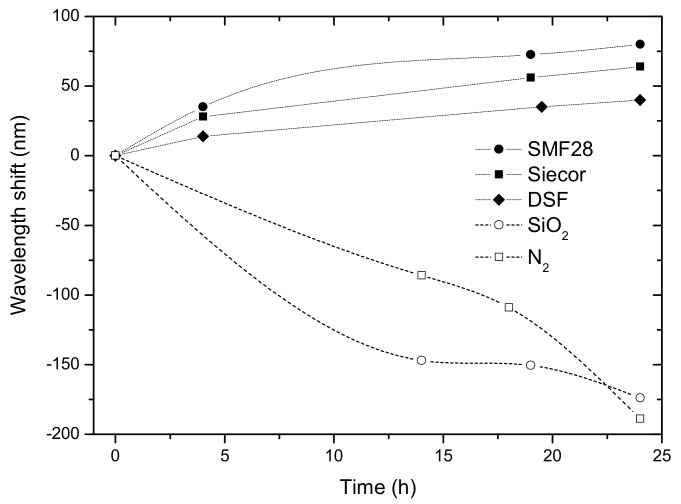
Time dependence of the resonant wavelength at 1000 °C. Reprinted with permission from ref. [97]. © 2021 IEE.

**Figure 9:**
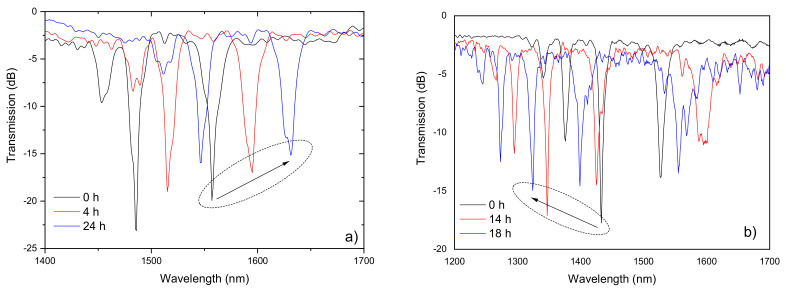
Evolution of the spectra of two gratings written in the (**a**) SMF28 fiber; and in a (**b**) nitrogen doped fiber during annealing at 1000 °C. Adapted with permission from ref. [97]. © 2021 IEE.

The thermal annealing of several gratings, inscribed in different type of fibers, at high temperatures was also investigated [97]. Figure 8 shows the wavelength shift during annealing performed at 1000 °C during 24 h. It is interesting to note that gratings inscribed in germanium doped fibers move towards longer wavelengths, while gratings inscribed in germanium free fibers (pure-silica-core and nitrogen doped fibers) the shift is much larger and is precisely in the opposite direction. There is no clear understanding of the reasons for this behavior. The standard fibers appear to approach an equilibrium situation, after a process of stress relaxation and thermal expansion, whilst for the other fibers diffusion and chemical processes might be involved leading to a degradation of the pristine fiber. As shown in Figure 9, gratings annealed at 1000 °C for 1 day exhibits some degree of degradation. A similar study was conducted by Morishita et al. [98] in the Corning SMF28 fiber. The shifts obtained are larger which might be due to the higher arc currents used in their work. It should be stressed that annealing at higher temperatures for a couple of hours wash out the grating (1100 °C) [98] followed by a degradation of the fiber itself (1200 °C, the annealing temperature approaches the fiber fictive temperature) [92].

**Figure 10:**
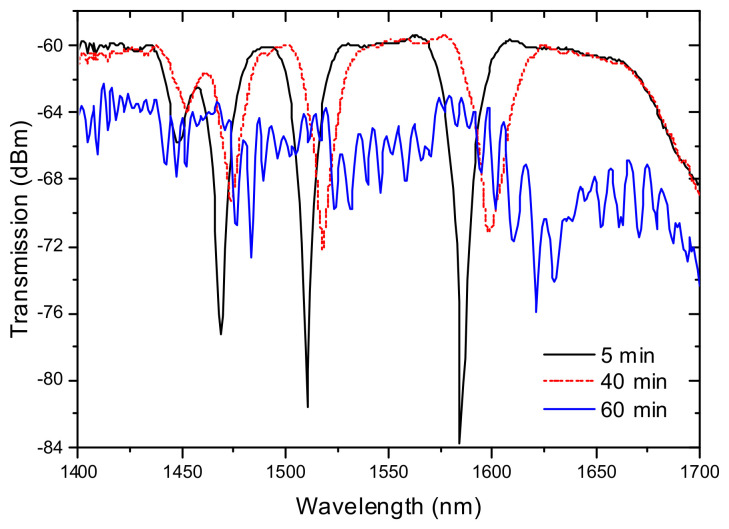
Annealing at 1190 °C of a 430 μm LPFG inscribed in the Corning DSF fiber. Reprinted from [86].

Figure 10 shows the evolution of the transmission spectra of a grating inscribed in the Corning DSF fiber during annealing at 1190 °C for 1 h [92]. The grating withstood 30 min with only minor changes, but it was erased after 1 h. The degradation has been related to the deformation of the fiber caused by the applied external tension (5.1 g) while the fiber was heated to a temperature in the limit of the softening range. In fact, it was later measured an elongation rate of ~6 mm/min for a 180 mm long SMF-28 fiber at 1300 °C under a tension of only 1.1 g.

**Figure 11:**
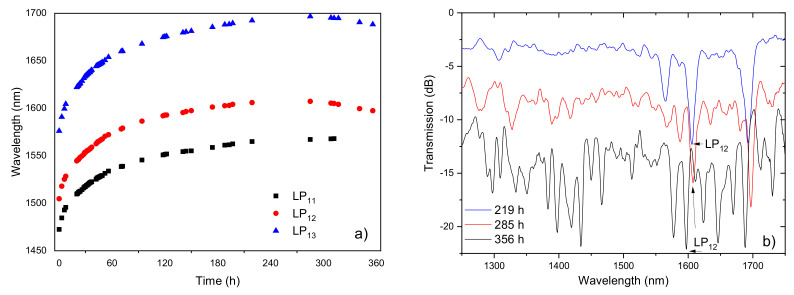
(**a**) Time dependence of the resonance wavelengths of three cladding modes and (**b**) evolution of the grating spectrum during the heat treatment at 1000 °C for two weeks. Adapted with permission from ref. [99]. © 2021 Elsevier.

A study on the long time, two weeks, annealing at 1000 °C was also conducted for a LPFG (5.1 g, 9 mA, 1 s, 540 μm, 40) inscribed in the SMF28 fiber [99]. It was observed a shift of the grating spectrum to longer wavelengths and a decrease of the resonances amplitude as a result of structural relaxations. For times longer than 200 h, it was found an increase of the background loss in the transmission spectrum and the appearance of the irregular wavelength dependence, which may be due to crystallization (Figure 11).

In conclusion, the use of arc-induced gratings as high temperature sensors requires proper annealing at a temperature (higher than the strain point) above the working temperature to avoid thermal hysteresis. For temperatures as high as 1000 °C, it should be avoiding the use of external pulling tensions to keep the fiber straight since it causes fiber elongation. For example, the fiber can instead, be inserted in a closed silica capillary, this would also mitigate the effect of devitrification that can be initiated by the presence of water vapor and oxygen at such high temperatures. In any case, its use should be limited in time since degradation of the waveguide conditions will occur in the limit, due to core dopants diffusion. Thus, the implementation of high temperature optical fiber sensors requires more stable materials such as sapphire.

### 2.5. LPFGs at Cryogenic Temperatures

**Figure 12:**
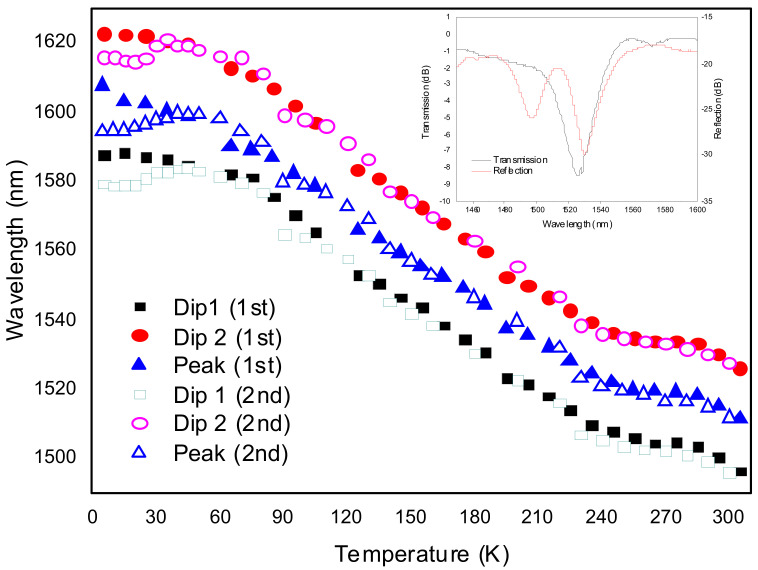
Wavelength of the two Dips and the Peak of the PS-LPFG, inscribed in the B/Ge co-doped fiber, as a function of temperature. Inset: PS-LPFG reflection spectrum. Adapted with permission from ref. [100]. © 2021 IEEE.

As important as the development of high temperature sensors is the development of sensors for cryogenic temperatures. We have investigated the thermal behavior of gratings arc-induced in the SMF28 fiber and also in the B/Ge co-doped fiber at cryogenic temperatures [100]. We concluded that the temperature sensitivity of gratings inscribed in the latter fiber is considerably higher. Our results are better than the ones obtained for fiber Bragg gratings, even for those embedded/bonded to substrates with very different thermal expansion coefficients and compares well with former works also using transmissive LPFGs in this kind of fiber [101,102]. Our phase-shifted LPFG works in reflection (the fiber was cleaved and polished near the grating and uses the Fresnel reflection of the end face) being an advantage for the implementation of practical sensors for extreme environments since it only requires access to one end of the fiber. Figure 12 shows the thermal behavior of the PS-LPFG from 5 K up to room temperature for two sets of experiments. The average temperature sensitivity obtained was −0.43 nm/K in the 60–240 K range. Values ranging from −0.08 nm/K up to 0.2 nm/K were obtained in the 5–35 K temperature range. Further studies are nevertheless required to understand the behavior at low temperatures.

## 3. LPFGs’ Sensitivity to Fabrication and Physical Parameters

### 3.1. Influence of the Fabrication Parameters on the Gratings Sensitivity

**Figure 13:**
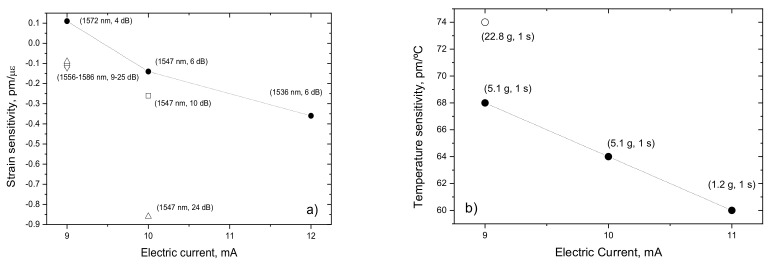
(**a**) Strain and (**b**) temperature sensitivity of LP_14_ for different values of electric current and external pulling tension. Reprinted with permission from ref. [103]. © 2021 IEE.

It is well-known that the sensitivity of long period gratings to changes in physical properties such as, temperature, strain and refractive index of the surrounding medium depends on the order of the cladding mode involved in the coupling [51,84,86]. This is a consequence of the dependence of the sensitivity equations on the slope of the matching curves for a particular resonance wavelength [84]. However, it was observed that changes in the fabrication parameters not only affect the position of the resonance wavelengths but also impact their sensitivities [103]. Figure 13a,b show the influence of the electric current and of the external pulling tension on the sensitivity to strain and temperature, respectively, of the LP_14_ cladding mode belonging to a 540 μm grating. It should be stressed that no systematic study was published so far. However, the obtained results demonstrate the ability to tune the strain and temperature sensitivity by 400% and 25%, respectively.

As discussed previously [104], depending on the value of the external pulling tension, the arc discharge can release or create new stresses in the optical fiber. It can also induce microdeformations in the fiber cross section. Therefore, the geometric and the structural changes will impact the effective refractive indexes of core and cladding modes and thus, from Equations (1)–(6), the grating sensitivity to both strain and temperature.

### 3.2. Sensitivity to Other Physical Parameters

**Figure 14:**
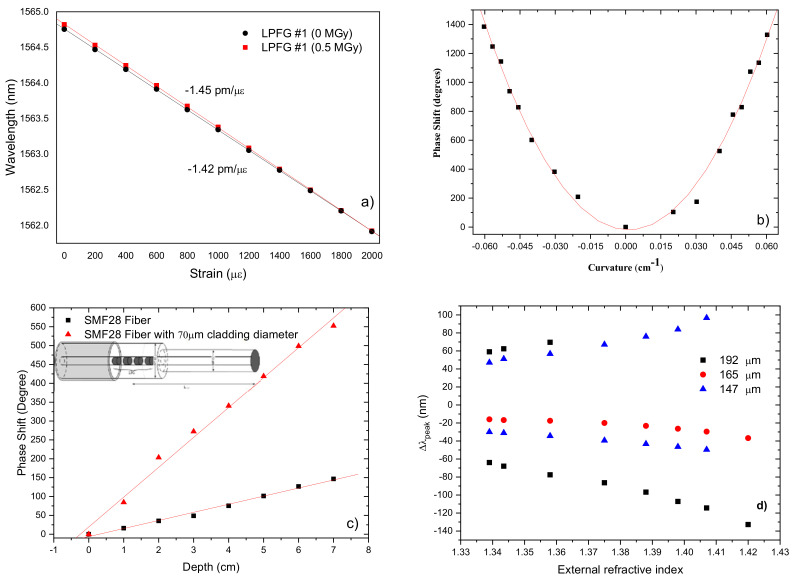
Characterization of LPFGs sensitivity to (**a**) Strain. Adapted with permission from [94] © 2021 The Optical Society; (**b**) bending. Reprinted with permission from [125]. © 2021 The Optical Society; (**c**) liquid level. Adapted with permission from [126] © 2021 SPIE; and (**d**) surrounding refractive index. Reprinted with permission from [127] © 2021 IEEE.

LPFGs are sensitive to other parameters such as strain [105,106,107,108], bending [109,110,111], torsion [112,113,114], pressure [115,116] and surrounding refractive index [89,117,118,119,120,121,122,123,124]. On contrary to what happens for the temperature sensitivity, the strain sensitivity is typically an order of magnitude lower than for FBGs. As discussed above, besides the order of the cladding mode the sensitivity increases with the decrease of the fiber cross section. However, a LPFG (36.3 g, 9 mA, 0.5 s, 730 μm, 75) inscribed in a pure silica core fiber exhibited a strain sensitivity of −1.4 pm/με (Figure 14a) [94] which compares well with the values obtained for FBGs and is also larger than the typical values shown in Figure 13. On the other hand, LPFGs are very sensitive to bending what is a considerable drawback when one wants to measure parameters other than curvature. Even though LPFGs can be used for bending [109] (Figure 14b), a Mach-Zehnder configuration comprising two LPFGs separated by 15 cm was used for refractive index measurement and it was demonstrated that the sensitivity can be improved if one bent the region in between the gratings [125]. LPFGs are also sensitive to the external refractive index and therefore they can be used as a liquid level sensor or a refractometric sensor [68,119]. Figure 14c present results on the behavior of a Michelson interferometer when different lengths of the cavity are immersed in water. The sensor sensitivity increases with the length of the cavity, the order of the cladding modes and also with the decrease of the cladding diameter [126]. Figure 14d presents the displacement of the resonance peaks as a function of the external refractive index for aqueous solutions. A sensitivity as high as −0.7 μm/RIU @1.33 was obtained for a 192 μm-LPFG near the turning points, inscribed in a B/Ge co-doped fiber [127]. The sensitivity can exceed 1.3 μm/RIU if the dual resonances are considered. These values are at least 20 times higher than the ones presented in [118]. In this context, further improvements have been achieved when gratings were inscribed in etched cladding fibers [122] and/or fibers were coated with thin films [79,128,129,130,131,132,133,134,135,136]. L. Coelho et al. at INESC TEC conducted relevant research related to the influence of metal oxide coating on arc-induced gratings and their applications as refractometric sensors [137,138,139,140,141,142,143,144,145].

### 3.3. Polarization Dependence Loss

**Figure 15:**
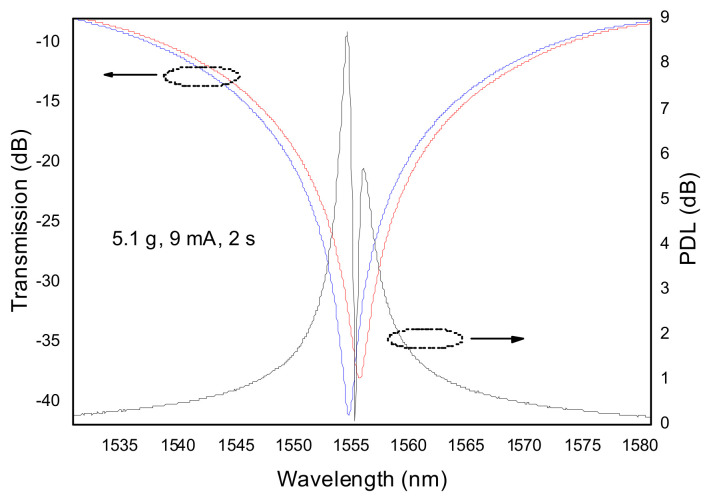
Transmission loss of LP_14_ resonance and respective PDL, inscribed using the following set of fabrication parameters: 5.1 g, 9 mA, 2 s and 30. Adapted with permission from ref. [81]. © 2021 Elsevier.

**Figure 16:**
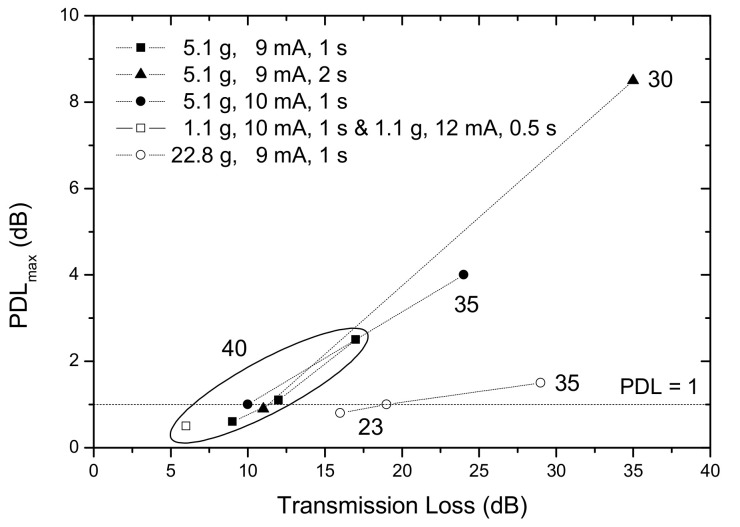
PDL values as a function of the transmission loss (LP_14_ cladding mode resonance) of several gratings produced under different fabrication parameters in the SMF28 fiber. Reprinted with permission from ref. [81]. © 2021 Elsevier.

In our fabrication setup the arc discharge is directional, originating a temperature gradient that will cause asymmetric microdeformations in the fiber cladding and core. Since the perturbation is asymmetric, coupling occurs to asymmetric cladding modes and the gratings are intrinsically birefringent, that is, they possess polarization dependence loss [81,87,146]. Figure 15 shows a resonance dip and the corresponding PDL for two orthogonal polarizations. The dip separation is of about 1 nm and the PDL value is near 9 dB. In general, PDL values increase with the increase of the resonance loss, that is, the larger the asymmetric perturbation the larger the PDL values. Lower values or no use of external pulling tension leads to lower PDL values. The same also occurs for symmetric perturbations. In addition to the use of π-shifted Sagnac loop interferometer [147], the rotation of the fiber by 180° between discharges was also suggested for PDL reduction [148]. By following the latter procedure results are in general better however, they are as good as for the conventional technique under certain conditions (namely, low pulling tension) (Figure 16). It is expected that further improvement may be achieved if the fiber is, prior to each discharge, rotated by 360/*n*, where *n* is the total number of arc discharges applied for the grating inscription.

### 3.4. Interactions with Uv-Radiation

**Figure 17:**
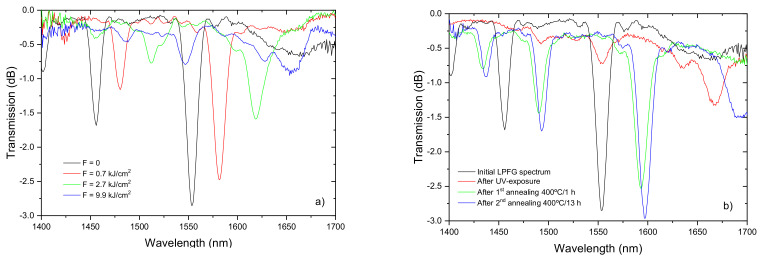
(**a**) Erasure of a LPFG arc-induced in a B/Ge co-doped fiber through uniform uv-radiation and (**b**) recover through thermal annealing. Reprinted with permission from ref. [96]. © 2021 Wiley Periodicals, Inc.

In the early days several studies were conducted in order to study the interactions between uv-radiation and arc discharges. In fact, initially we would prefer to have knowledge on the changes that an arc discharge would cause to a fiber. For instance, we could make successive discharges along the fiber and afterwards write a fiber Bragg grating over it. The displacement of the Bragg wavelength in comparison to the one in a pristine fiber would give information on the core refractive index change caused by the arc discharge. Moreover, by writing tilted-FBGs on the pristine and arc-treated fiber, it is also possible to acquire information on the cladding refractive index change [149,150]. However, sooner was realized that the results would depend on the characteristics of the arc, the type of fiber, hydrogen loaded fiber and temperature annealing. In any case it was observed that after exposure of arc-induced gratings to uniform uv-radiation the spectrum would be almost completely erased. Afterwards if the fiber was properly annealed the spectrum would be almost fully recovered, although the resonance dips would be located at longer wavelengths. The change was attributed to a reaction of the hydrogen with the germanium doped silica matrix during the first annealing above 100 °C. It is interesting to note that for both, Ge-doped and B/Ge co-doped fibers, the spectrum was recovered when a temperature coincident to the strain point was reached, 700 °C and 400 °C, respectively [96]. At this temperature starts the relaxation of elastic stresses and as will be discussed below that impacts the movement of the resonance dips. Nevertheless, the overall displacement towards longer wavelengths was larger than expected for just stress relaxation which means that some permanent refractive index change was caused by the uv-radiation. Figure 17 shows the evolution of the spectrum of a LPFG arc-induced in a B/Ge co-doped fiber while exposed to uniform uv-radiation and its afterwards recover while submitted to thermal annealing at 400 °C.

We have also demonstrated that a sampled FBG can be produced by fabricating a uv-induced FBG on top of an arc-induced grating and such a device was used to perform the simultaneous measurement of temperature and strain [151]. On the other hand, arc discharges can be applied to create, among other devices, apodised FBGs and Fabry-Perot filters [152].

### 3.5. Exposure to γ-Radiation

**Figure 18:**
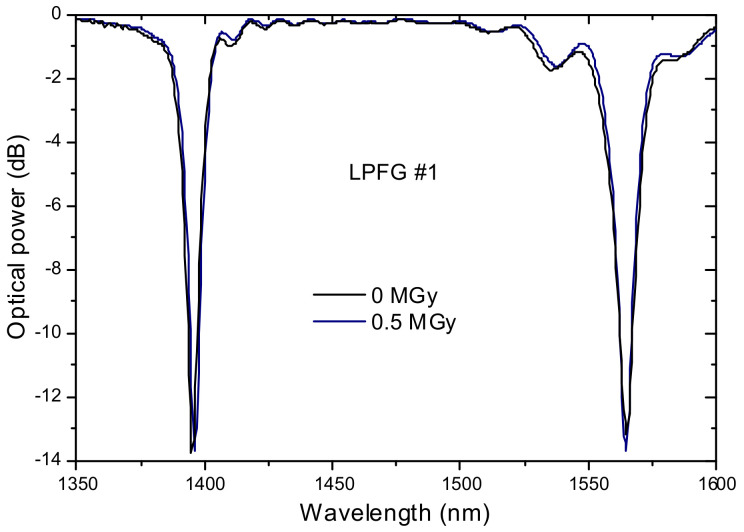
Effect of *γ*-radiation on an LPFG arc-induced in a pure-silica-core fiber. Adapted with permission from ref. [94]. © 2021 The Optical Society.

The effect of gamma radiation on arc-induced grating was also investigated [94]. It was demonstrated that total doses up to 0.5 MGy does not induces changes in the gratings spectra neither in their temperature and strain sensitivities (Figure 18). These results enable the possibility to use LPFGs arc-induced in pure-silica-core fibers, known as being a radiation-resistant optical fiber, for structural health monitoring of nuclear energy facilities.

Recently, there is an increasing interest for this topic, since gratings arc-induced in non-radiation-resistant fibers can be used as radiation dosimeters [153,154,155,156,157].

## 4. Long-Period Fiber Gratings as Optical Fiber Sensors

In this section we will present three sensors for the simultaneous measurement of temperature and strain and also a flow sensor, based on the use of a LPFG or LPFG/FBG. As mentioned above LPFGs are very sensitive to physical parameters such as bending and in general there is cross sensitivity between two or more physical parameters. For instance, when measuring strain, the shift of the resonance wavelengths is also affected by temperature changes. Therefore, it is important to implement sensors able to measure simultaneously more than one parameter. This can be easily achieved by monitoring more than one resonance of a single LPFG since they exhibit different sensitivities to physical parameters. In general, the higher the order of the cladding mode resonance the higher the sensitivity. The problem is that resonances can be hundreds of nanometers away from each other and, therefore, two optical sources are required. There are several ways to overcome the issue, for instance, by using gratings with different periods having close resonances or having a grating inscribed in two dissimilar fibers. We have proposed three different approaches that enable the fabrication of a compact sensor in a single fiber requiring a single optical source: a sampled FBG, a step-changed LPFG and a dual LPFG inscribed in a B/Ge co-doped fiber. All schemes show neighbor resonances having different sensitivities to temperature and strain.

### 4.1. Sampled Fiber Bragg Grating

**Figure 19:**
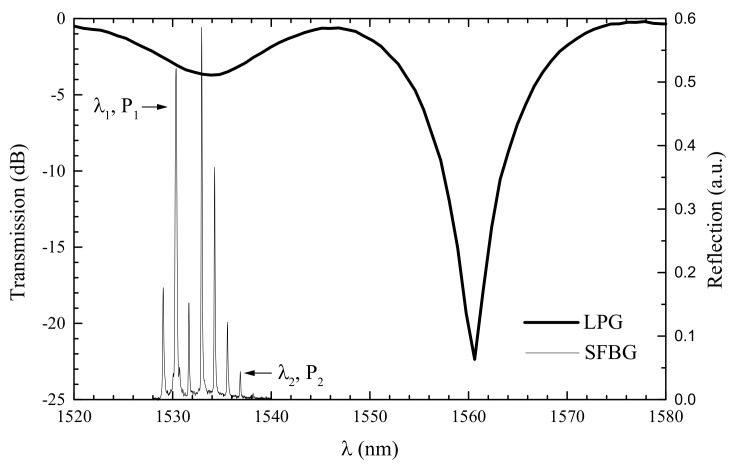
LPFG transmission spectrum and reflection spectrum of the sampled-FBG. Reprinted with permission from [151]. © 2021 IEE.

This sensor comprises the fabrication of a UV-induced FBG on top of an arc-induced LPFG [151]. After the inscription in a Corning dispersion-shifted fiber of a 25 mm-long LPFG with a period of 619 μm, the fiber was hydrogen loaded. Subsequently a 10 mm-long FBG was written over the LPFG using 248 nm radiation from a KrF laser through a uniform phase mask with a period of 538 nm. Since the arc discharge periodically modifies the core effective refractive index, the result is the growth of a sampled FBG exhibiting different resonance wavelengths [158]. Figure 19 shows the transmission and reflection spectrum of this sensor. As it can be seen the spectrum of the sampled FBG possess resonance wavelengths that stand in the different slopes of a higher order resonance of the LPFG. Since the temperature and strain sensitivity of both gratings is different, it was possible to convert the wavelength changes in optical power changes. Thus, a cheaper sensor head was developed, where optical power fluctuations were also compensated, and presented rms resolutions of ±0.32 °C and ±5.4 με. As a final remark, it should be mentioned that a systematic study is still open since the behavior of this assembly depend not only on the relative physical location of both gratings but also on the location of their resonance wavelengths.

### 4.2. Step-Changed LPFG

**Figure 20:**
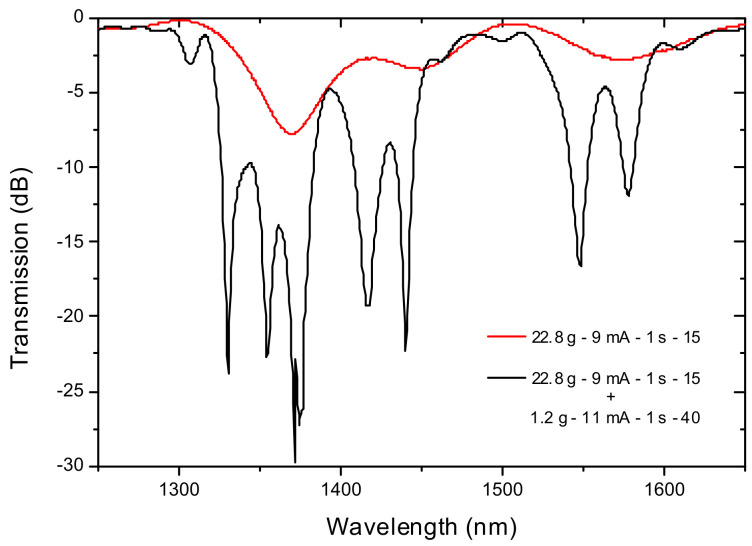
Evolution of the grating spectrum during the fabrication spectrum. Adapted from [86].

Another interrogation scheme makes use of the fact that fabrication parameters of arc-induced gratings change not only the position of the resonance wavelengths but also their sensitivity. Thus, a first section of a 540 μm-LPFG was inscribed with the following fabrication parameters: T = 22.8 g, I = 9 mA, t = 1 s, N = 15 and afterwards they were modified during the inscription of the second section: T = 1.2 g, I = 11 mA, t = 1 s, N = 40, without any physical separation (Figure 20). This procedure resulted in a step-changed LPFG in which the spectrum exhibits two neighbour resonances in the third telecommunication window with different sensitivities to changes in temperature and strain, respectively 60.0/74.2 pm/°C and −0.373/0.0 pm/με. The *rms* deviations corresponding to temperature and strain were found to be ±0.2 °C and ±35 με, respectively [159].

### 4.3. Dual Set of Resonances Corresponding to Different Formation Mechanisms

**Figure 21:**
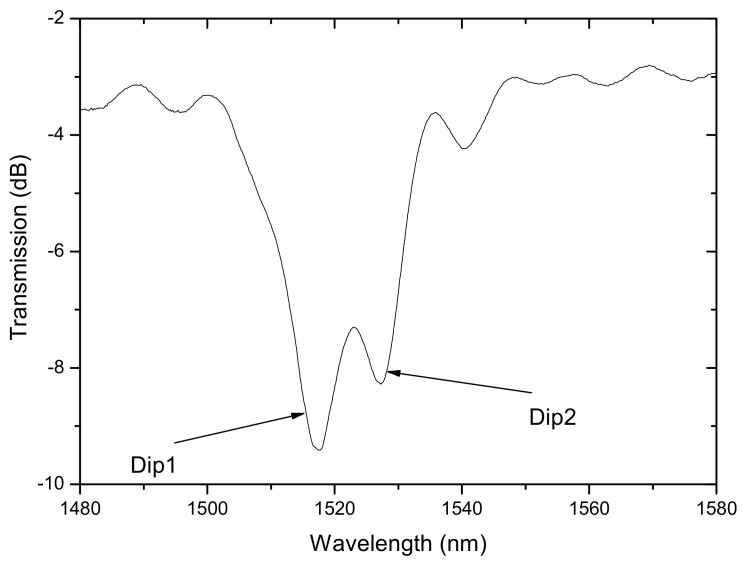
Dual resonances induced by different mechanisms in the B/Ge co-doped fiber. Reprinted with permission from [113]. © 2021 The Optical Society.

**Figure 22:**
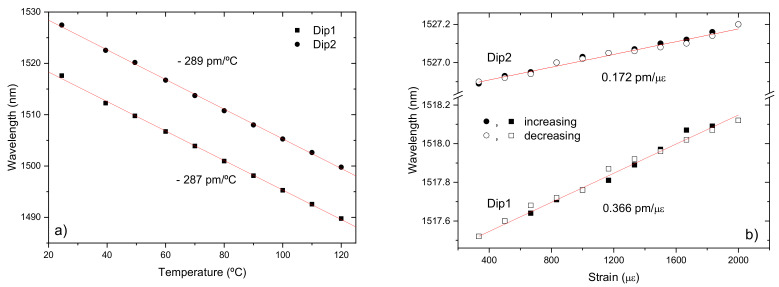
(**a**) Temperature and (**b**) strain sensitivities. Reprinted with permission from [113]. © 2021 The Optical Society.

We have also proposed a new sensor based on the fact that under certain writing conditions it is possible to obtain in the B/Ge co-doped fiber a dual set of resonances belonging to distinct gratings. In fact, in this kind of fibers, LPFGs are typically arc-induced due to a densification mechanism being the coupling to symmetric cladding modes. However, when the arc discharge is directional and an external pulling tension is used during the grating inscription, the mechanism of formation in a standard fiber is due to microdeformations. Thus, by placing the fiber in a region where the temperature gradient is higher and the average temperature is lower we were able to increase the asymmetric mechanism to a level comparable to the symmetric one. Therefore, the transmission spectrum shows to neighbor resonances being the one at longer wavelengths due to densification and the other, at shorter wavelengths, due to microdeformations (Figure 21). We have demonstrated that through thermal annealing at high temperatures that the resonance at longer wavelengths vanishes [160]. The temperature and strain sensitivities of these resonances are, respectively, −287/−289 pm/°C and 0.366/0.172 pm/με (Figure 22). Therefore, this sensor can perform temperature-compensated strain measurements.

### 4.4. Flow Sensor

**Figure 23:**
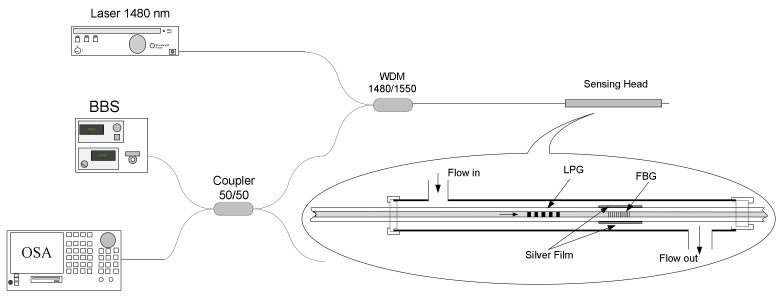
Experimental setup with detail of the sensing head. Reprinted with permission from [161]. © 2021 The Optical Society.

**Figure 24:**
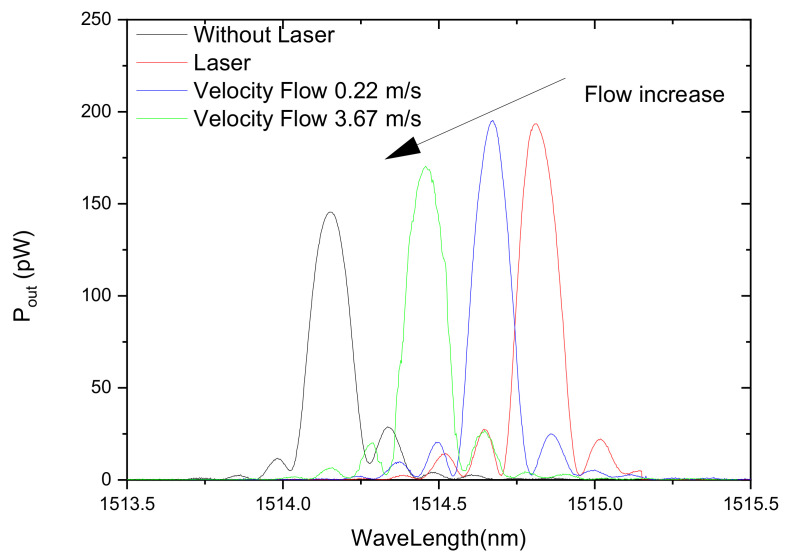
Response of the FBG Bragg wavelength as a function of air flow (laser on). Adapted with permission from [161]. © 2021 The Optical Society.

The measurement of fluid velocity or flow is very important for instance to determine potential leaks of medicine gases in distribution pipes existing in health facilities. We have proposed an elegant flow sensor head comprising two gratings: a LPFG and a metal coated FBG [161]. The physical principle relies on heat transfer mechanisms, namely, convection. Basically, radiation from a pump laser, with 400 mW output power at 1480 nm, is injected into the fiber and reaches a 385 μm-LPFG, arc-induced in the SMF28 fiber, which exhibits a coincident resonance wavelength. The radiation rejected by the resonance is afterwards absorbed by a 15 mm-long uniform silver thin film that overcoats a 10 mm-long FBG, with a Bragg wavelength centered at 1514 nm. A broadband source centered at 1550 nm is used to illuminate both gratings (Figure 23). Thus, as heat is absorbed by the film, its temperature increases and, therefore, the grating signature shifts towards longer wavelengths. Being the sensor head inserted in a test channel, air with increasing velocity flows through the channel and removes heat from the film, essentially, through convection and the Bragg wavelength shifts towards shorter wavelength following the decrease in temperature. The convection heat transfer coefficient depends on the fluid velocity and therefore a proper calibration enables the sensor to work as a flowmeter (Figure 24).

## 5. Conclusions

We have reviewed the most important achievements related to the properties and applications of arc-induced long-period fiber gratings. The thermal behavior of gratings was studied and some conclusions were drawn: gratings arc-induced in B/Ge co-doped fibers may find application at cryogenic temperatures due to their high temperature sensitivity; on the other hand, arc-induced gratings require proper annealing in order to be used at temperatures up to 1000 °C; long term use at high temperatures is limited due to potential fiber elongation, crystallization and core dopants diffusion. Gratings arc-induced in pure-silica-core fibers may find application in the monitoring of structural health in nuclear facilities. Interactions between arc discharges and uv-radiation enable the fabrication of several devices and to acquire knowledge on the changes caused by the discharges to the optical fiber. The polarization dependence loss was analyzed and identified as an intrinsic property that constitutes a drawback for most applications in optical communication domain. It was shown that the fabrication parameters can be used to tune the sensitivity of arc-induced gratings to changes in physical parameters. Several applications of LPFGs as sensors, namely, for the simultaneous measurement of temperature and strain and also as flowmeters were presented. Arc-induced gratings may also find application as refractometric sensors based on the use of gratings in the turning points coated with thin films in the transition region.

## Figures and Tables

**Table 1:** Temperature sensitivity of gratings written in different fibers. Adapted from [86].

Fiber (mol% GeO_2_)	Dcore/μm	*Λ*/μm	Mode Order	Temperature Sensitivity (pm °C^−1^)
Sumitomo_1.5	8.3	540	4	58.1 + 0.125 T
SMF-28_3	8.6	540	4	72.3 + 0.12 T
Siecor_6	8.3	540	4	76.7 + 0.11 T
HI-980	~4	540	5	56.2 + 0.09 T
Corning_DSF_12	5.1	540	3	39.4 + 0.13 T
Al	4.25	400	4	64.7 T ≤ 700
Al/Er	4.55	400	4	60.1 T ≤ 700
S	not available	540	4	50 T ≤ 700
Oxford_SiO_2_/F	9	730	2	47.1 T ≤ 300
ACREO_SiO_2_/F	not available	730	3	40.1 T ≤ 300
N’94	2	240	6	45 T ≤ 900
N’96	4.5	540	5	43 T ≤ 900
N1905	6.2	400	4	67 T ≤ 400
N1940	5.8	400	4	51.9 T ≤ 400
